# BDKRB1 activation induces CXCR2 desensitization in neutrophils during severe sepsis and exacerbates disease severity

**DOI:** 10.1172/jci.insight.185743

**Published:** 2025-12-08

**Authors:** Raquel Duque do Nascimento Arifa, Carolina Braga Resende Mascarenhas, Lívia Caroline Resende Rossi, Maria Eduarda Freitas Silva, Larissa M. Lucas, João Paulo Pezzini Barbosa, Daiane Boff, Brenda Gonçalves Resende, Lívia Duarte Tavares, Alesandra Corte Reis, Vanessa Pinho, Flavio Almeida Amaral, Caio Tavares Fagundes, Cristiano Xavier Lima, Mauro Martins Teixeira, Daniele G Souza

**Affiliations:** 1 Laboratory of Microorganism-Host Interaction, Department of Microbiology, Universidade Federal de Minas Gerais (UFMG), Belo Horizonte, Brazil.; 2 Instituto René Rachou, FIOCRUZ-Minas, Belo Horizonte, Brazil.; 3 Department of Morphology and; 4 Department of Biochemistry and Immunology, Institute of Biological Sciences, Belo Horizonte, Brazil.; 5 Department of Surgery, Medical School, UFMG, Belo Horizonte, Brazil.

**Keywords:** Inflammation, Microbiology, Bacterial infections, Cell migration/adhesion, Neutrophils

## Abstract

Sepsis is a life-threatening organ dysfunction caused by a dysregulated host response to infection. During early sepsis, kinins are released and bind to B1 (BDKRB1) and B2 (BDKRB2) bradykinin receptors, but the involvement of these receptors in sepsis remains incompletely understood. This study demonstrated that the genetic deletion of *Bdkrb2* had no significant impact on sepsis induced by cecal ligation and puncture (CLP) compared to wild-type (WT) mice. In contrast, *Bdkrb1^−/−^* mice subjected to CLP exhibited decreased lethality and bacterial load, associated with an increased influx of neutrophils into the peritoneal cavity, compared with WT mice. Neutrophils from CLP-*Bdkrb1^−/−^* mice partially restored CXCR2 expression and reduced the upregulation of P110γ observed in WT CLP neutrophils. Pharmacologic inhibition of BDKRB1 combined with imipenem treatment substantially improved survival compared with antibiotic therapy alone. In human neutrophils, stimulation with LPS led to the upregulation of BDKRB1 expression, and antagonism of BDKRB1 restored neutrophil migration in response to CXCL8. These findings identify BDKRB1 as an important modulator of neutrophil dysfunction in sepsis and a promising therapeutic target whose inhibition improves bacterial clearance, restores neutrophil migration, and increases the efficacy of antibiotic treatment.

## Abstract 2

BDKRB1 activation contributes to sepsis-induced hyperinflammation and controls the activation of PI3Kγ and the expression of CXCR2 on neutrophils, impairing their migration during the disease.

## Graphical abstract

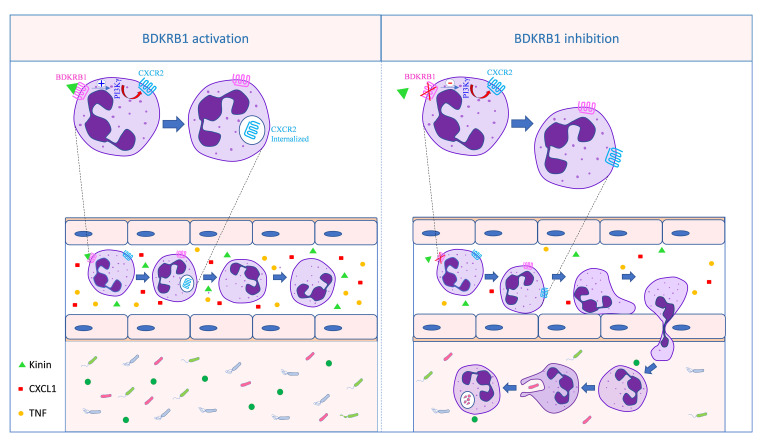

## Introduction

Sepsis is a potentially fatal organ failure resulting from unbalanced host response to infection (1) and includes an earlier stage known as systemic inflammatory response syndrome (SIRS). In SIRS, elevated pro-inflammatory cytokines and widespread accumulation of inflammatory cells in vital organs can lead to organ failure, tissue damage, and death. Neutrophils, the first line of defense against infection (2, 3), rely on the activation of CXCR2 to effectively migrate to the site of infection (4). However, in sepsis, overproduction of pro-inflammatory mediators and increased activation of pattern recognition receptors trigger PI3Kγ, leading to phosphorylation of G protein–coupled receptor kinases (GRKs), which can desensitize CXCR2 on neutrophils (4). Reduced CXCR2 expression together with overexpression of CCR2 impairs neutrophil migration to the site of infection and favors their accumulation in the microvasculature of various organs, leading to multiple organ dysfunction syndrome (2, 3).

Bradykinin-related peptides are vasoactive molecules that play a role in various inflammatory conditions, including infection-related inflammation (5–7). These peptides are produced by kallikreins acting on kininogens and exert their effects via the activation of 2 G protein–coupled receptors: B1 and B2 bradykinin receptors (BDKRB1 and BDKRB2) (8). BDKRB2 is constitutively expressed in various tissues and regulates several physiological processes (9). In contrast, BDKRB1 is only sparsely expressed in healthy tissues but is upregulated in response to inflammatory mediators, such as TNF and IL-1β (8, 10). However, the role of BDKRB1 in the migration of leukocytes to sites of infection and its importance in sepsis are still poorly understood. The aim of this study was to investigate whether activation of BDKRB1 and BDKRB2 contributes to the inability of neutrophils to migrate to the site of infection in sepsis. The results show that BDKRB1 activation impedes CXCR2-mediated accumulation of neutrophils at the site of infection, resulting in impaired bacterial clearance, organ dysfunction, and sepsis-related mortality.

## Results

### BDKRB1, but not BDKRB2, plays a pivotal role in the pathogenesis of severe polymicrobial sepsis.

**Figure 1:**
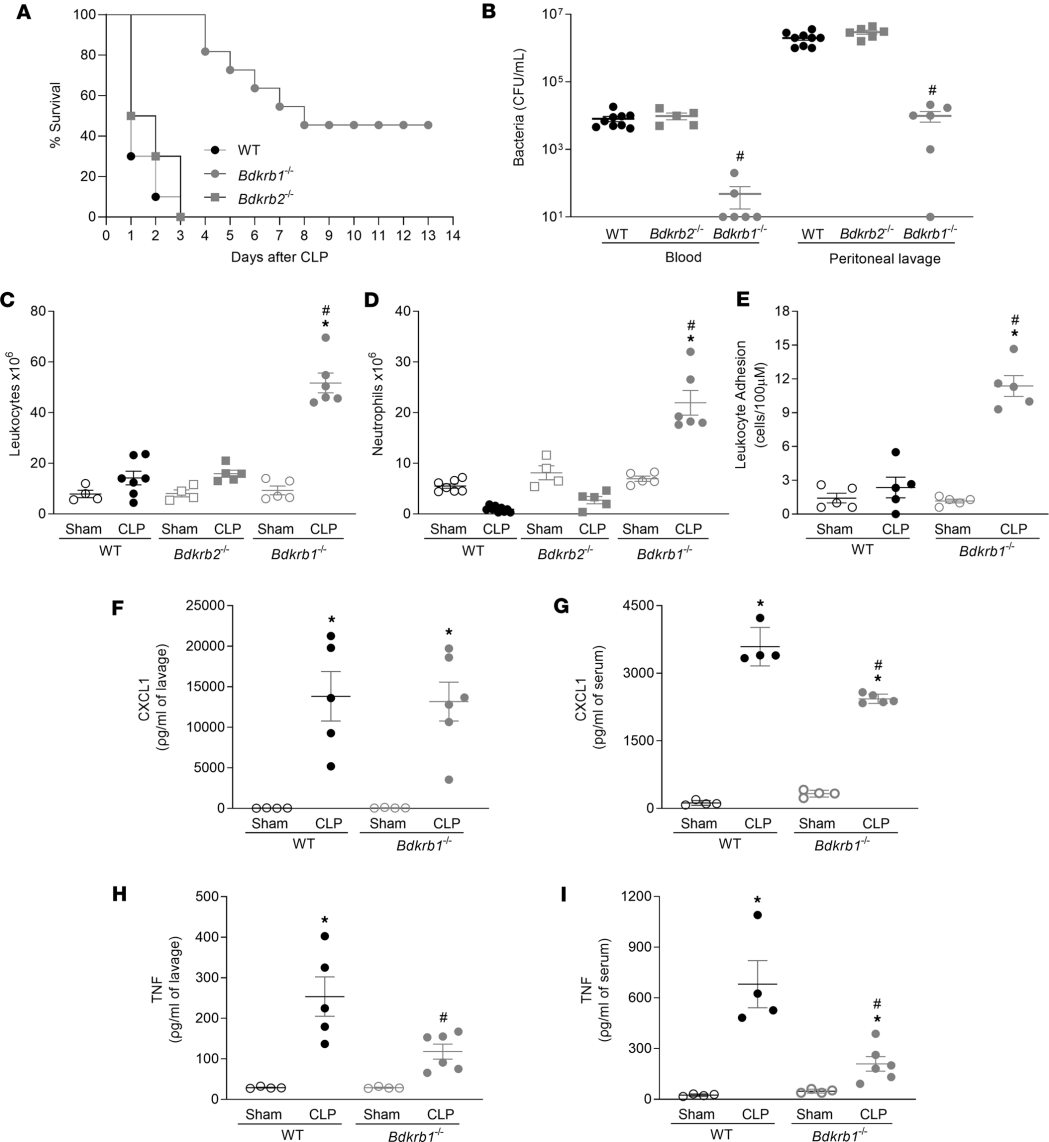
BDKRB1, but not the BDKRB2, plays a pivotal role in the pathogenesis of CLP sepsis. WT, *Bdkrb1^−/−^*, or *Bdkrb2^−/−^* mice were subjected to sham or CLP sepsis surgery. (**A**) Survival of mice subjected to CLP sepsis was followed for 14 days. Results are percentage of (**A**) survival, analyzed by the log-rank (Mantel-Cox) test, with 10 mice/group. Six hours after CLP sepsis, groups of mice were euthanized, blood was drawn, and peritoneal lavage was performed. (**B**) CFUs in blood and peritoneal exudate, (**C**) total leukocytes, and (**D**) neutrophil recruitment in the peritoneal cavity were analyzed. (**E**) Leukocyte adhesion was analyzed 3 hours after CLP in the cremaster by intravital microscopy. Results are expressed as mean ± SEM of at least 4 animals/group. Six hours after CLP, groups of mice were euthanized, blood was drawn, and peritoneal lavage was performed. (**F** and **G**) CXCL1 and (**H** and **I**) TNF concentrations were quantified in the peritoneal lavage and serum. Experiments were replicated at least once, and 1 representative experiment is presented. Survival analysis was performed with at least 10 animals/group. The Mantel-Cox log-rank test (χ^2^) was used for statistical analysis of survival curves. ANOVA followed by Tukey’s multiple comparisons posttest was used for data with a normal distribution. For data that did not follow a normal distribution, the Kruskal-Wallis test followed by Dunn’s multiple comparisons posttest was applied to compare 3 or more independent groups. *P* < 0.05. *, *P* < 0.05, compared with the sham group; ^#^, *P* < 0.05, compared with the WT mice subjected to CLP.

In the severe sepsis model, 100% of WT or *Bdkrb2*^−/−^ mice were dead 3 days after cecal ligation and puncture (CLP). In contrast, *Bdkrb1*^−/−^ mice began to die after 4 days and 50% were still alive on day 7 after CLP (Figure 1A). Six hours after CLP, the bacterial load recovered in blood and peritoneal lavage was comparable in WT and *Bdkrb2*^−/−^ mice but about 2 logs lower in *Bdkrb1*^−/−^ mice (Figure 1B). In septic WT and *Bdkrb2*^−/−^ mice, migration of leukocytes, especially neutrophils, into the peritoneal cavity was markedly restricted, whereas in *Bdkrb1*^−/−^ mice there was a marked influx of neutrophils (Figure 1, C and D). In WT mice, the inability of neutrophils to reach the site of infection was associated with reduced adherence to the endothelium, which was not the case in *Bdkrb1*^−/−^ mice (Figure 1E). Thus, activation of BDKRB1 impedes neutrophil migration and consequently compromises bacterial clearance in the CLP model, whereas BDKRB2 appears to be less important under these conditions. Therefore, *Bdkrb2*^−/−^ mice were not used for further experiments.

CLP led to increased concentrations of CXCL1 and TNF in the peritoneal lavage and serum (Figure 1, F–I) of WT mice. Except for TNF, *Bdkrb1*^−/−^-CLP mice showed increased cytokine levels compared with *Bdkrb1*^−/−^-sham mice in the peritoneal lavage (Figure 1, F and H). Interestingly, sepsis induced in *Bdkrb1*^−/−^ mice resulted in lower concentrations of CXCL1 in serum and of TNF in both peritoneal lavage and serum compared with WT-CLP mice (Figure 1, F–I). CLP also led to increased CXCL2, IL-1β, and IL-10 concentrations in the lavage fluid of both WT and *Bdkrb1*^−/−^ mice, though there was no substantial difference between the WT and *Bdkrb1*^−/−^ mice subjected to CLP (Supplemental Table 1; supplemental material available online with this article; https://doi.org/10.1172/jci.insight.185743DS1). CLP also induced an increase in IL-6 production in the peritoneal lavage, but it is noteworthy that this cytokine concentration was reduced in *Bdkrb1*^−/−^ mice (Supplemental Table 1).

**Figure 2:**
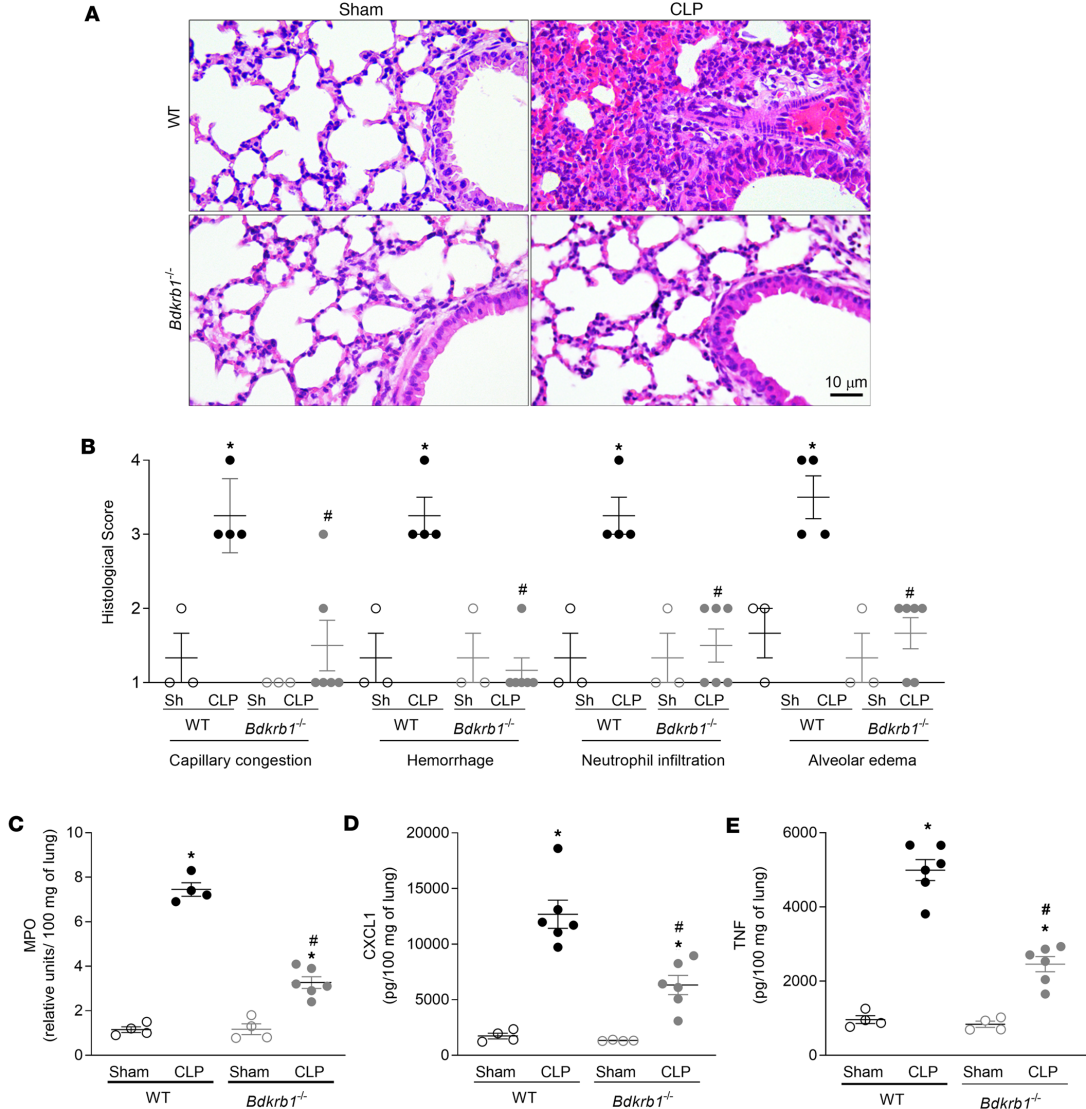
Absence of BDKRB1 prevents lung inflammation and injury. (**A**) Lung sections were stained with H&E for histopathologic analysis, and (**B**) tissue damage was quantified in 3 sections of each sample to assess capillary congestion, hemorrhage, neutrophil infiltration, and alveolar edema. (**C**) Neutrophil influx was determined by quantification of MPO activity. (**D**) CXCL1 and (**E**) TNF concentration in the lung was determined by ELISA. Experiments were replicated at least twice, and 1 representative experiment is presented in the figure. Results are expressed as mean ± SEM of at least 4 animals per group. ANOVA followed by Tukey’s multiple comparisons posttest was used for data with a normal distribution. For data that did not follow a normal distribution, the Kruskal-Wallis test followed by Dunn’s multiple comparisons posttest was applied to compare 3 or more independent groups. *, *P* < 0.05, compared with the sham group; ^#^, *P* < 0.05, compared with WT mice subjected to CLP.

CLP induced marked lung injury characterized by severe capillary congestion, hemorrhage, infiltration of inflammatory cells, and alveolar edema (Figure 2, A and B) and increase in neutrophil influx into the lungs, as assessed by myeloperoxidase (MPO) activity (Figure 2C) and histological analysis (Figure 2, A and B). *Bdkrb1*^−/−^ mice showed improvement in all histopathologic parameters (Figure 2, A and B), resulting in a substantial reduction in lung injury and MPO activity (Figure 2C) compared with WT mice. The lungs of CLP-WT mice showed an intense production of cytokines, as shown by the increase in CXCL1 (Figure 2D) and TNF (Figure 2E). Indeed, CLP led to an increase in these cytokines in the lungs of *Bdkrb1*^−/−^ mice compared with the sham group. However, the levels of CXCL1 (Figure 2D) and TNF (Figure 2E) decreased in CLP-*Bdkrb1*^−/−^ mice compared with CLP-WT mice. The production of CXCL2, IL-1β, IL-6, and IL-10 in the lungs of WT and *Bdkrb1*^−/−^ mice was increased. Interestingly, the concentrations of CXCL2 and IL-6 were reduced in *Bdkrb1*^−/−^ mice compared with WT mice (Supplemental Table 1). These results suggest that BDKRB1 plays a central role in CLP-induced lung inflammation and injury.

### BDKRB1 in myeloid cells plays an essential role in exacerbating the inflammatory response triggered by severe sepsis.

**Figure 3:**
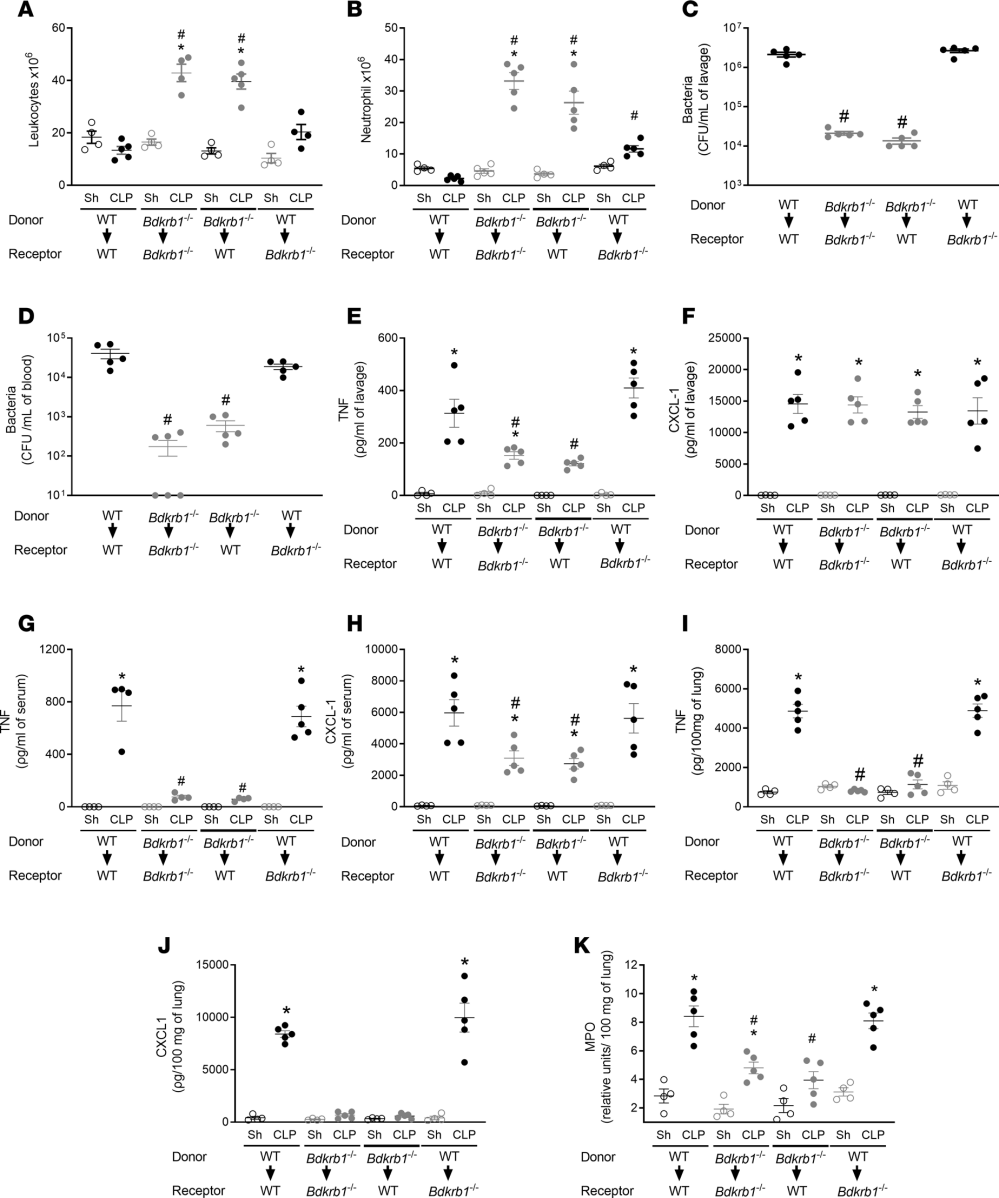
BDKRB1 in myeloid cells plays an essential role in exacerbating the inflammatory response induced by CLP. Chimeric mice were generated by the adoptive transfer of 10^6^ BM cells from donor to recipient mice. Four groups of mice were generated: cells from WT mice to WT mice, cells from *Bdkrb1^−/−^* mice to *Bdkrb1^−/−^* mice, cells from *Bdkrb1^−/−^* mice to WT mice, and cells from WT mice to *Bdkrb1^−/−^* mice. Each group was divided into 2 groups that underwent sham or CLP surgery. Six hours after CLP, the groups of mice were euthanized, blood and lungs were collected, and peritoneal lavage was performed. (**A**) Total leukocytes and (**B**) neutrophil influx into the peritoneal cavity. (**C**) The bacterial load in peritoneal exudate and (**D**) blood was determined by plating and CFU counting. The concentrations of (**E**) TNF and (**F**) CXCL1 were determined in peritoneal lavage, (**G** and **H**) blood, and (**I** and **J**) lungs. (**K**) MPO activity was determined in the lung. Experiments were replicated at least twice, and 1 representative experiment is presented in the figure. Results are expressed as mean ± SEM of at least 5 animals per group. ANOVA followed by Tukey’s multiple comparisons posttest was used for data with a normal distribution. For data that did not follow a normal distribution, the Kruskal-Wallis test followed by Dunn’s multiple comparisons posttest was applied to compare 3 or more independent groups. *, *P* < 0.05, compared with the sham group; ^#^, *P* < 0.05, compared with WT mice subjected to CLP.

To investigate the role of BDKRB1 in myeloid cells in CLP sepsis, *Bdkrb1*^−/−^ and WT mice were irradiated and subsequently transplanted with bone marrow–derived cells (BMDCs) from *Bdkrb1*^−/−^ or WT mice. WT mice receiving *Bdkrb1*^−/−^ BMDCs (*Bdkrb1*^−/−^ *→* WT) exhibited increased peritoneal leukocyte (Figure 3A) and neutrophil (Figure 3B) counts and reduced local (Figure 3C) and systemic (Figure 3D) bacterial load after CLP compared with *Bdkrb1*^−/−^ mice transplanted with WT BMDCs (WT *→* *Bdkrb1*^−/−^). Furthermore, *Bdkrb1*^−/−^ *→* WT mice exhibited similar cytokine levels and neutrophil influx into the lungs as *Bdkrb1*^−/−^ mice receiving *Bdkrb1*^−/−^ BMDCs (*Bdkrb1*^−/−^ *→* *Bdkrb1*^−/−^) (Figure 3, B and E–K). In contrast, *Bdkrb1*^−/−^ mice receiving BMDCs from WT (WT*→* *Bdkrb1*^−/−^) showed the same phenotype as WT mice receiving WT BMDCs (WT*→* WT), no neutrophil migration (Figure 3, B and K), and consequently a high bacterial burden (Figure 3, C and D) after CLP. In addition, WT-*Bdkrb1*^−/−^ mice had higher concentrations of TNF and CXCL1 in the peritoneal cavity (Figure 3, E and F), blood (Figure 3, G and H), and lungs (Figure 3, I and J) compared with *Bdkrb1*^−/−^-WT mice. These results emphasize that the role of BDKRB1 in sepsis depends on its presence in myeloid cells, which are critical for the migration in neutrophils to the site of infection and for the regulation of the inflammatory response CLP triggers.

### BDKRB1 activation induces increased PI3Kγ expression and desensitization of CXCR2 on neutrophils.

**Figure 4:**
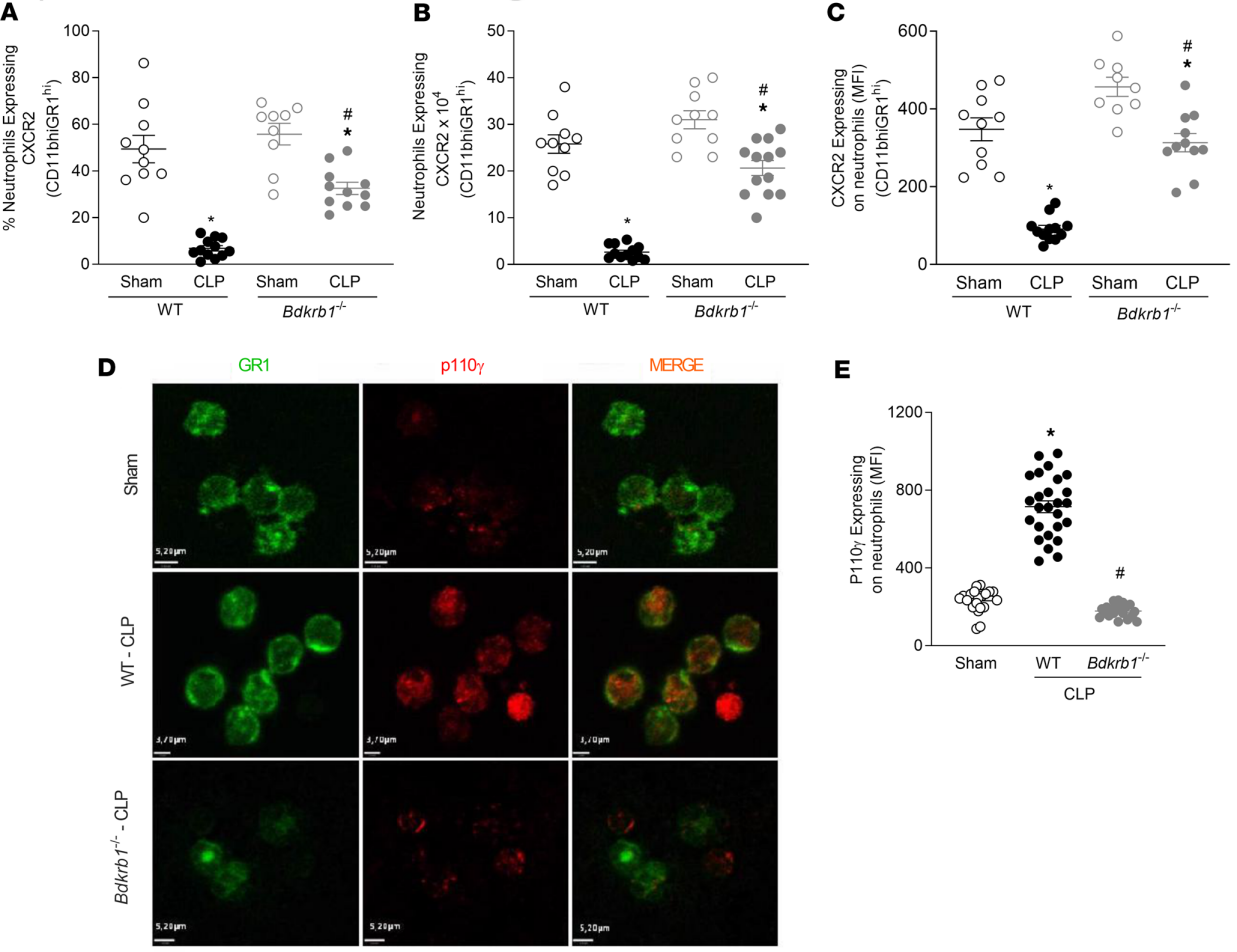
BDKRB1 activation induces PI3Kγ activation and desensitization of CXCR2 in circulating neutrophils after CLP-induced sepsis. Two hours postsurgery, circulating neutrophils from WT and *Bdkrb1*^–*/*–^ mice subjected to CLP or sham procedures were analyzed. (**A**) Percentage of neutrophils positive for CXCR2 labeling. (**B**) Number of circulating neutrophils expressing CXCR2. (**C**) MFI of CXCR2 in neutrophils, assessed by flow cytometry. (**D**) Representative immunofluorescence images showing neutrophils labeled with anti-Gr1 (green), P110γ labeled with anti-P110γ (red), and the merged overlay. Scale bar: 5.2 µm. (**E**) Quantification of P110γ immunofluorescence (MFI), measured across 20 cells per group. Data are representative of at least 2 independent experiments. ANOVA followed by Tukey’s multiple comparisons posttest was used for data with a normal distribution. For data that did not follow a normal distribution, the Kruskal-Wallis test followed by Dunn’s multiple comparisons posttest was applied to compare 3 or more independent groups. *P < 0.05 compared with the sham group and ^#^*P* < 0.05 compared with the WT CLP group.

CLP induced a decrease in both the percentage (Figure 4A) and number of circulating neutrophils (Figure 4B) expressing CXCR2 in WT mice, accompanied by a decrease in the MFI of CXCR2 on these cells (Figure 4C). Furthermore, CLP-induced sepsis resulted in upregulation of P110γ, the catalytic subunit of PI3Kγ, in WT mice compared with WT-sham mice (Figure 4, D and E). The genetic absence of BDKRB1 resulted in a reversal of CXCR2 desensitization and P110γ activation, which were very similar to the profiles observed in sham-operated mice. Collectively, these results suggest that BDKRB1 disrupts neutrophil migration by desensitizing CXCR2 in response to PI3Kγ activation.

**Figure 5:**
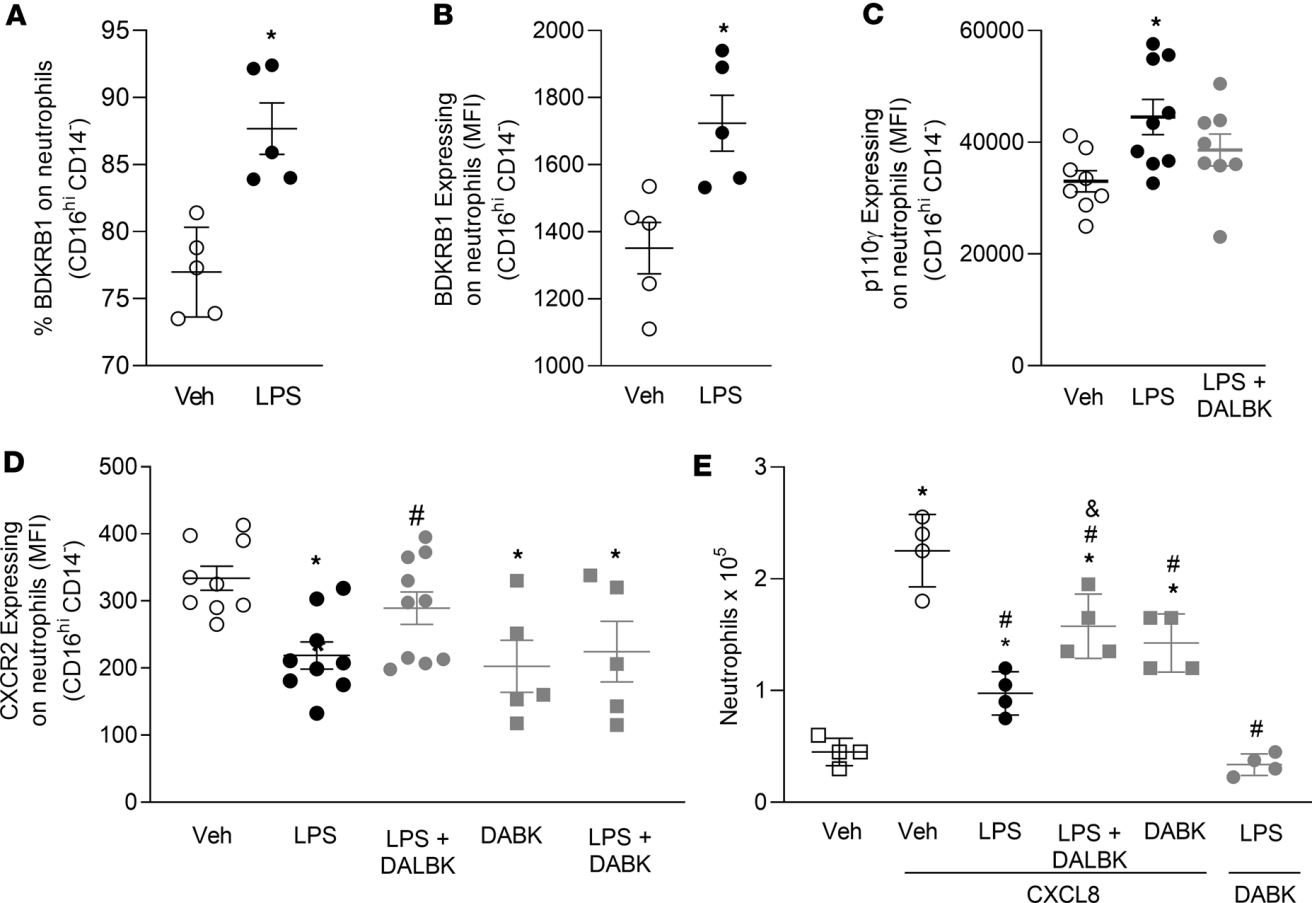
Activation of BDKRB1 impairs the migratory capacity of human neutrophils under septic conditions. Human neutrophils isolated from healthy donor blood were stimulated with LPS (10 μM) or vehicle control for 2 hours. Panels show (**A**) percentage of neutrophils positive for BDKRB1; MFI of (**B**) BDKRB1, (**C**) P110γ, and (**D**) CXCR2. (**E**) Transwell migration assay: cells pretreated for 30 minutes with BDKRB1 agonist Des-Arg^9^-Bradykinin (DABK, 1 μM) or antagonist des-Arg^9^[Leu^8^]-Bradykinin (DALBK, 10 μM), then allowed to migrate toward CXCL8 (10 μM) or DABK (1 μM, included as a chemotactic control). Data are presented as mean ± SEM from ≥ 5 wells per group for panels **A**–**D** and from 4 wells per group for panel **E**. *P < 0.05 versus control; ^#^*P* < 0.05 versus LPS-treated group for panels **A**–**D**. *P < 0.05 versus vehicle control; ^#^*P* < 0.05 versus vehicle + CXCL8–treated group; ^&^*P* < 0.05 versus LPS + CXCL8–treated group for panel **E**. The Mann-Whitney *U* test was used to assess the significance of differences between the means of 2 groups. ANOVA followed by Tukey’s multiple comparisons posttest was used for data with a normal distribution. For data that did not follow a normal distribution, the Kruskal-Wallis test followed by Dunn’s multiple comparisons posttest was applied to compare 3 or more independent groups. All experiments were replicated at least twice, and 1 representative experiment is shown.

Stimulation of human neutrophils with LPS resulted in increased percentage of BDKRB1-expressing cells (Figure 5A) and higher expression of BDKRB1, as assessed by the MFI (Figure 5B). Preincubation with LPS also resulted in upregulation of P110γ expression (Figure 5C). Interestingly, treatment with DALBK, a selective BDKRB1 antagonist, inhibited the LPS-induced increase in P110γ levels. To further investigate the role of BDKRB1, we treated neutrophils with LPS or DABK, a specific BDKRB1 agonist, and examined CXCR2 expression. Both LPS and DABK independently decreased neutrophil CXCR2 expression; however, their combined treatment did not result in an additive effect. Of note, DALBK reversed the LPS-induced downregulation of CXCR2, highlighting the involvement of BDKRB1 in modulating expression (Figure 5D). Consistent with these results, pretreatment with LPS or DABK reduced neutrophil transmigration in response to CXCL8, whereas DALBK restored migration impaired by LPS. Moreover, DABK failed to induce neutrophil migration after LPS exposure (Figure 5E).

### BDKRB1 antagonist improves sepsis and has a synergistic effect with broad-spectrum antibiotics.

**Figure 6:**
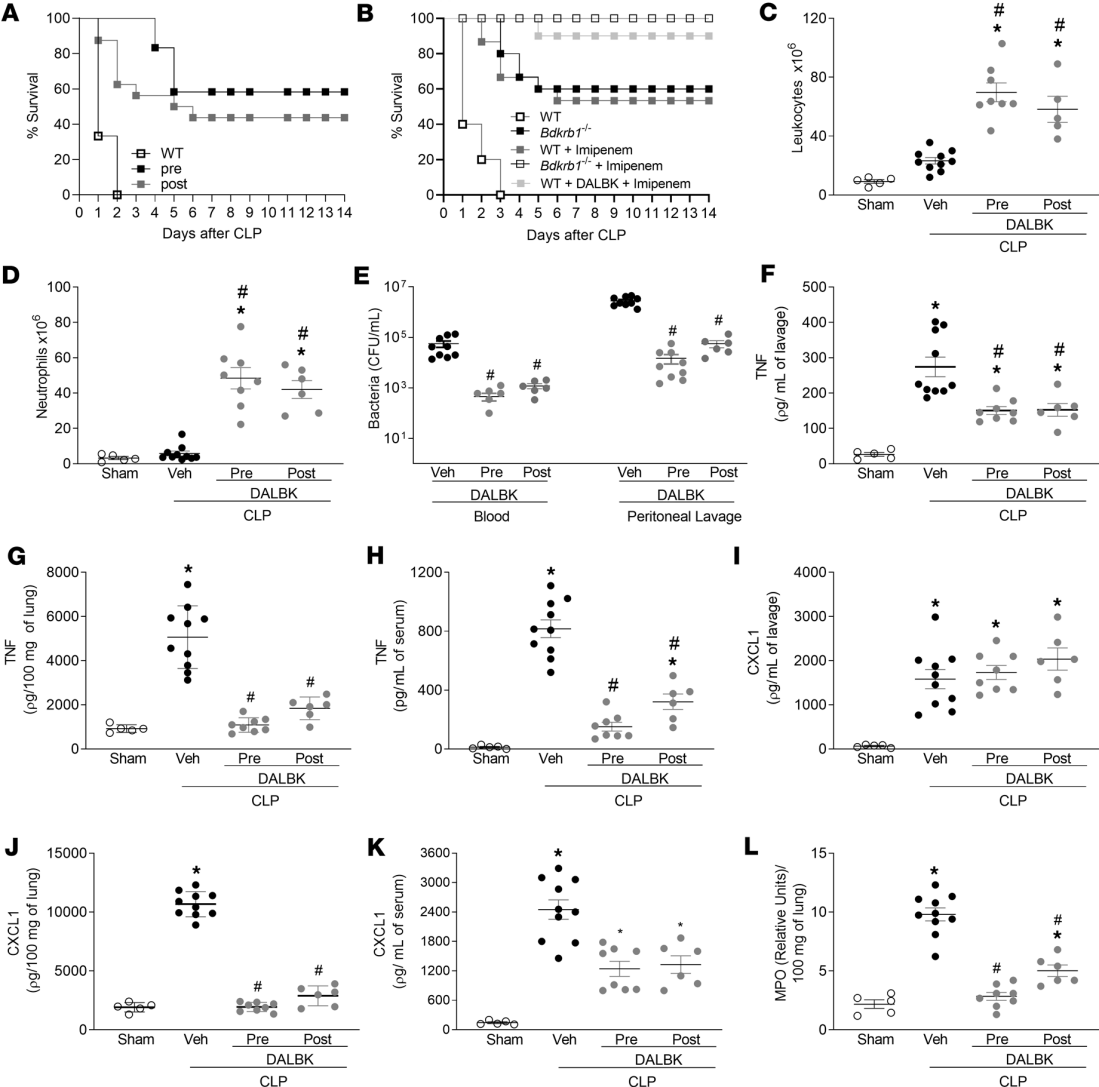
Treatment with a BDKRB1 antagonist prevents neutrophil dysfunction, bacterial burden, inflammation, and lethality associated with CLP in WT mice. (**A**) Survival of WT mice treated with a BDKRB1 antagonist alone and (**B**) survival of mice treated post-CLP with both a BDKRB1 antagonist and the antibiotic imipenem, monitored for 14 days. Imipenem was administered 3 hours after sepsis induction. Survival rates (%) were analyzed using the log-rank (Mantel-Cox) test; *n* = 10 mice per group. WT mice received the BDKRB1 antagonist DALBK (50 nM/kg) either 1 hour before (pretreatment) or 3 hours after (posttreatment) CLP. Six hours after CLP, mice were euthanized, and blood, lungs, and peritoneal lavage fluid were collected. (**C**) Total leukocyte count, (**D**) neutrophil influx into the peritoneal cavity, and (**E**) bacterial load (CFU) were evaluated. Concentrations of TNF were measured in the (**F**) peritoneal lavage, (**G**) lung, and (**H**) serum. CXCL1 levels were measured in the (**I**) peritoneal lavage, (**J**) lung, and (**K**) serum. (**L**) MPO activity was assessed in lung tissue. Survival analysis was performed with at least 10 animals per group. The Mantel-Cox log-rank test (χ^2^) was used for statistical analysis of survival curves. ANOVA followed by Tukey’s multiple comparisons posttest was used for data with a normal distribution. For data that did not follow a normal distribution, the Kruskal-Wallis test followed by Dunn’s multiple comparisons posttest was applied to compare 3 or more independent groups. Results are expressed as mean ± SEM from at least 5 animals per group. *P < 0.05 vs. sham group; ^#^*P* < 0.05 vs. WT-CLP group. All experiments were replicated at least twice; 1 representative experiment is shown.

WT mice subjected to CLP were treated with DALBK, a BDKRB1 antagonist, 1 hour before (pretreatment) or 6 hours after (posttreatment) the CLP procedure. Both pre- and posttreatment DALBK reduced CLP-induced lethality. Approximately 50% of mice survived up to 14 days after sepsis (Figure 6A). Since broad-spectrum antibiotics are the recommended treatment for patients with sepsis, we next examined the effect of treatment with DALBK in mice previously treated with imipenem. Figure 6B shows that imipenem, as well as DALBK alone, reduced CLP-induced lethality by approximately 50%. Interestingly, posttreatment DALBK provided 90% protection against lethality in CLP mice that had previously been treated with an antibiotic. *Bdkrb1*^−/−^ mice treated with antibiotics survived 100% of the time. In addition, both pretreatment and posttreatment DALBK prevented the lack of migration of neutrophils into the peritoneal cavity (Figure 6, C and D), resulting in a reduction of bacterial load in the peritoneal lavage and serum (Figure 6E) compared with vehicle treatment. Pre- and posttreatment DALBK substantially reduced the TNF concentration in the peritoneal cavity, serum, and lung (Figure 6, F–H) induced by CLP. In addition, pre- or posttreatment DALBK reduced CXCL1 concentration in lung and serum (Figure 6, J and K), though it had no effect on CXCL1 concentration in the peritoneal cavity (Figure 6I). BDKRB1 blockade also reduced CLP-induced MPO activity in the lung (Figure 6L). Importantly, taken together, these results suggest that DALBK could be a potential agent for the treatment of sepsis.

## Discussion

Despite numerous advances in the use of antimicrobials and resuscitation therapies for sepsis (11), the mortality rate remains high, and new therapeutic strategies for the treatment of this disease are urgently required. This study shows that (a) BDKRB1 plays an essential role in the pathogenesis of sepsis, at least in part by BDKRB1 impairing neutrophil migration to the infection site (peritoneal cavity) while promoting neutrophil migration to remote sites (such as the lungs) during the disease; (b) BDKRB1 exerts its effect in myeloid cells by controlling the activation of P13Kγ and the expression of CXCR2; and (c) BDKRB1 has beneficial effects as an adjunct to antibiotic therapy, highlighting DALBK as a potential adjuvant treatment for sepsis.

Previously, Ruiz et al. showed that a BDKRB1 antagonist reduced lethality, lung and kidney injury, and vascular permeability in a CLP-induced septic shock model. Here, we demonstrated that BDKRB1 antagonism or its absence reduces lethality and lung injury by increasing neutrophil recruitment to the infection site and decreasing their accumulation in the lungs. The reduction in neutrophils is associated with decreased tissue injury in several experimental models, such as improved wound healing (12). The infection site during CLP sepsis is the peritoneal cavity, and neutrophil migration to the infection site is driven by activation of CXCR2, which is downregulated during sepsis. On the other hand, CCR2 is upregulated during sepsis, promoting inappropriate migration of neutrophils to remote organs, such as lung, increasing the tissue injury in these organs (2, 13, 14). Our CLP experimental model reproduced this phenomenon. Several studies demonstrated that BDKRB1 activation can contribute to neutrophil adhesion, chemotaxis, and migration (15, 16). However, in our sepsis conditions, the BDKRB1 contributes to the failure in neutrophil migration to the infection site, one phenomenon that is also observed related to CXCR2 activation. In the absence of BDKRB1, neutrophil migration to the peritoneal cavity is restored, and the influx of neutrophils into the lungs is reduced. This improved neutrophil migration facilitated bacterial clearance and reduced systemic cytokine production and mortality. BDKRB2 blockade had no effect on local inflammation, bacterial burden, or mortality.

BDKRB1 expressed on neutrophils (17) can induce chemotaxis (15), enhance the expression of integrin (16), and trigger the release of matrix metalloproteinases (MMP9) and MPO in a PI3K- and PKC-dependent manner in human neutrophils (18). Our study showed that the absence of BDKRB1 limits the overproduction of cytokines. Previous results show that the absence of BDKRB1 reduces TNF production in the intestine, lung, and serum during intestinal ischemia and reperfusion in mice (6). Lower cytokine concentrations are associated with lower lethality. Under our experimental conditions, blocking BDKRB1 results in a more efficient, self-limiting inflammatory response that allows clearance of pathogens without excessive tissue damage. Our data are consistent with the findings of Nasseri and colleagues, who showed that the BDKRB1 antagonist BI113823 reduced the expression of NF-κB and COX-2 in the lungs of mice following LPS-induced lung injury, resulting in reduced lung injury (19). This study also showed that the same antagonist reduced cytokine concentrations and ameliorated lung injury in a polymicrobial sepsis rat model. Our results showed that the role of BDKRB1 in sepsis appears to be related to its presence in BMDCs, as demonstrated using chimeric mice.

Activation of BDKRB1 appears to be associated with the internalization of CXCR2 in circulating neutrophils during severe sepsis. BDKRB1-deficient mice exhibited increased CXCR2 surface expression. This was associated with decreased expression of P110γ, a subunit of PI3Kγ, which is involved in the internalization of CXCR2 (20). Inhibition of PI3Kγ resulted in downregulation of GRK2 expression, thereby reducing CXCR2 internalization, which contributed to increased CXCR2 expression on neutrophils and improved survival in septic mice (20). In summary, the absence or inhibition of BDKRB1 prevents PI3Kγ upregulation, increases CXCR2 expression, and enhances neutrophil influx to the peritoneal cavity. Our results therefore suggest that kinins acting on BDKRB1 trigger PI3Kγ activation, leading to CXCR2 internalization. Although we have clearly shown that CLP sepsis induces an increase in P110γ in neutrophils and that the absence of the BDKRB1 reverses this increase, our data do not allow us to conclude that this activation occurs directly through the agonist of the BDKRB1, since these changes could occur through other indirect mechanisms, such as a reduced cytokine production. Additional studies are necessary to elucidate this mechanism. Alternatively, kinins may reduce neutrophil migration through NO production (19). NO triggers the activation of soluble guanylate cyclases (sGCs) as well as the formation of cGMP and the phosphorylation of PKG (21). Inhibition of sGC and PKG during sepsis improved survival by increasing CXCR2 expression and neutrophil migration. These results shed light on the complex mechanisms that influence neutrophil responses in sepsis.

Our results also show that activation of BDKRB1 is sufficient to modulate CXCR2 expression in human neutrophils, even in the absence of LPS stimulation. Both LPS and DABK, a selective BDKRB1 agonist, independently reduced CXCR2 expression, and their combination did not result in an additive effect. This suggests that the activation of LPS and of BDKRB1 converge in a common signaling pathway. Furthermore, the use of DALBK, a BDKRB1 antagonist, reversed the LPS-induced downregulation of CXCR2, emphasizing the role of BDKRB1 in this regulatory mechanism. These findings are consistent with previous studies showing that LPS promotes the expression of BDKRB1 (22) and increases the production and release of kallikreins in neutrophils (23). Neutrophils can produce tissue kallikrein (23), as well as other kininogenases, such as elastase and proteinase 3, which can process plasma-derived high and low molecular weight kininogens that bind to the neutrophil membrane (24, 25). The resulting kinins, such as bradykinin, can then be converted by neutrophil membrane-bound carboxypeptidase M into DABK, a selective agonist of BDKRB1 (26). Taken together, our data suggest that BDKRB1 activation, whether by LPS-induced endogenous kinin production or by exogenous DABK, leads to downregulation of CXCR2 on neutrophils, likely through PI3Kγ signaling.

Moreover, blocking BDKRB1 enhanced the efficacy of antibiotic therapy. Combining antibiotics with modulators of the immune response is a promising approach, but previous agents showed limited results (20, 27). Treatment with anti-TNF or IL-1Ra has not had a positive effect on mortality in patients with sepsis (28), suggesting that new candidates are urgently needed. The inhibition of BDKRB1 improves neutrophil migration and bacterial clearance, making it a valuable therapeutic candidate for the treatment of sepsis. In summary, our research highlights BDKRB1 as a promising therapeutic target in sepsis. Its inhibition not only modulates the inflammatory response and improves bacterial clearance but also enhances the efficacy of antibiotic treatment. This synergistic effect makes BDKRB1 blockade a potential complementary strategy for the treatment of sepsis.

## Methods

### Sex as a biological variable.

All animals used in this study were male. This choice was made to minimize variability in early mortality, as CLP is a severe model associated with high lethality, and male mice have been shown to exhibit greater resistance to this procedure. The use of male animals ensured more consistent outcomes under severe CLP conditions.

### Animals.

*Bdkrb1*^−/−^ (29) and *Bdkrb2*^−/−^ (30) mice were generated as previously described. C57BL/6J-WT mice were purchased from the Biotério Central of the UFMG. Eight- to 12-week-old mice were housed in separate cages under standard conditions and had free access to commercial food and water. The experiments were previously approved by the Animal Ethics Committee of the UFMG (Protocols 137/2012 and 136/2014).

### Experimental model of severe polymicrobial sepsis induced by CLP.

Mice underwent either severe sepsis by CLP with an 18-gauge needle (CLP) or laparotomy only (sham control) as previously described (31). Survival of mice was monitored for 14 days after CLP. Mice were euthanized 6 hours after sepsis to collect peritoneal lavage, blood, and lung samples.

### Migration of leukocytes to the peritoneal cavity.

Total leukocytes were counted using the Neubauer chamber. Differential count was performed according to morphological characteristics. Quantification of the individual cell types was determined by the percentage of counted cells in relation to the total number of cells.

### Bacterial count.

Peritoneal lavage and blood samples were plated on Petri dishes containing Mueller-Hinton agar medium (Difco Laboratories). After 24 hours of incubation at 37°C, the colonies were counted, and the results were expressed as the logarithm (log10) of CFU per milliliter.

### MPO assay.

Neutrophil activity in the lung was determined by measuring MPO activity as previously described (32). Lung samples were frozen in liquid nitrogen and later processed to determine MPO activity. The change in optical density was measured at 450 nm using tetramethylbenzidine (Sigma-Aldrich). Results were expressed in relative units, comparing MPO activity with that of casein-stimulated mouse peritoneal neutrophils processed in a similar manner.

### Cytokine measurement.

The concentrations of TNF, IL-6, IL-1β, IL-10, CXCL1, and CXCL2 were determined in the intraperitoneal lavage and in lungs; TNF and CXCL1 were determined in the serum. Cytokine concentrations were determined by ELISA using commercially available kits from R&D Systems.

### Histopathological analysis.

The 5 μm–thick lung sections were stained with hematoxylin and eosin. Lesions such as alveolar congestion, hemorrhage, neutrophil infiltration, aggregates in alveoli or vessel walls, and thickening of the interalveolar septum were identified and scored on a scale from 0 (absent) to 4 (severe) to standardize the assessment (33).

### Isolation of neutrophils from blood and chemotaxis assay.

Separation of human neutrophils (about 90% purity) was performed using the double-density histopaque method and quantified by trypan blue method. Chemotaxis assays were performed using a Modified Boyden chamber (Neuroprobe) and polycarbonate filters (4 μm pores; Neuroprobe) as previously described (34). A total of 4 × 10^5^ neutrophils/mL were incubated with LPS (10 μM) or vehicle for 2 hours. The BDKRB1 agonist, DABK (1 μM), and the BDKRB1 antagonist, DALBK (10 μM), were then added for an additional 30 minutes. Cells were labeled for flow cytometry analysis. For the chemotaxis assay, CXCL8 (10 μM) or DABK (1 μM) was placed on the bottom of the plate while the neutrophil suspension was placed on the cell culture inserts. After 60 minutes at 37°C, neutrophils that migrated to the bottom of the plate were counted in a Neubauer chamber.

### Chimera protocol.

WT or *Bdkrb1*^−/−^ mice were exposed to γ radiation of 9 Gy with Co60 source in a Gammacell 220 irradiator. Bone marrow cells from WT or *Bdkrb1*^−/−^ mice were transplanted intravenously into the irradiated recipient mice on the same day. Transplantation groups included WT-WT, *Bdkrb1*^−/−^-*Bdkrb1*^−/−^, *Bdkrb1*^−/−^-WT, and WT-*Bdkrb1*^−/−^ (donor-recipient, respectively). After cell injection, the mice received ciprofloxacin in their drinking water (70 mg/L) for 15 days. After a 10-day resting phase, mice were subjected to CLP sepsis. Schulze-Topphoff and colleagues demonstrated that the degree of chimerism with *Bdkrb1*-deficient bone marrow cells exceeds 95% (35).

### Intravital microscopy.

Four hours after sepsis induction the rolling and adhesion of leukocytes to the mesenteric vascular endothelium were examined according to established procedures (36). Leukocyte rolling was quantified as the number of rolling leukocytes per minute per vessel, and adhesion of leukocytes was confirmed when they remained on the vein endothelium for at least 30 minutes.

### Immunofluorescence.

Mouse neutrophil purification was performed using the double-density histopaque method. The isolated neutrophils were subjected to the cytospin method and stained using the Panotic technique. The purity of the neutrophils was above 90%, allowing for subsequent immunofluorescence analysis. The experiment was conducted 2 hours after surgery on mice. Neutrophils from WT-CLP or sham and *Bdkrb1^−/−^* CLP or sham mice were isolated and labeled with anti-GR1 FITC (catalog 108405, clone RB6-8C5, BioLegend) and primary anti-P110γ (catalog SC-7177, clone H-199, Santa Cruz Biotechnology) and secondary anti-rabbit IgG PE (catalog IC1051P, clone 60024B, R&D Systems) antibodies to evaluate the fluorescence profile of P110γ in neutrophils. P110γ was quantified using MFI. The MFI of P110γ was determined for 20 cells from each group of mice.

### Flow cytometry.

One hundred microliters of mouse blood was incubated with anti-CD45 Pacific Blue (catalog 103125, clone 30-F11, BioLegend), anti-CD11b PerCP-Cy5.5 (catalog 101227, clone M1/70), anti-GR1 FITC (catalog 108405, clone RB6-8C5, BioLegend), and anti-CXCR2 PE (catalog 149303, clone SA044G4, BioLegend). Neutrophils were identified by sequential gating on SSC-A versus FSC-A, FSC-H versus FSC-A (single cells), CD45^+^ cells, and then CD11b^hi^GR1^hi^ cells. Human neutrophils isolated from healthy donor blood were stimulated with LPS (10 μM) or vehicle control for 2 hours, followed by an additional 1-hour incubation with DABK or DALBK. Cells were then stained with anti-CD14 AF488 (catalog 367115, clone 63D3, BioLegend), anti-CD16 PE (catalog 302008, clone 3G8, BioLegend), anti-BDKRB1 (catalog SC-25484, clone J2912, Santa Cruz Biotechnology), anti-CXCR2 (catalog FAB331P, clone 48311, R&D Systems), anti-P110γ (catalog SC-7177, clone H-199, Santa Cruz Biotechnology), and the corresponding secondary antibodies: anti-rabbit IgG AF488 (catalog IC1051G, clone 60024B, R&D Systems) and anti-rabbit IgG PE (catalog IC1051P, clone 60024B, R&D Systems). Human neutrophils were identified by gating on SSC-A versus FSC-A, FSC-H versus FSC-A (single cells), and selecting CD16^hi^CD14^–^ cells. Flow cytometric analysis was performed using a BD FACSCanto II instrument, and data were analyzed with FlowJo software.

### Statistics.

Results were expressed as mean ± SEM for groups of 4 to 10 animals, and all experiments were repeated twice, yielding consistent results. One-way ANOVA followed by Tukey’s multiple comparisons posttest was used for data with a normal distribution. For data that did not follow a normal distribution, the Kruskal-Wallis test followed by Dunn’s multiple comparisons posttest was applied to compare 3 or more independent groups. The Mann-Whitney *U* test was used to assess the significance of differences between the means of 2 groups. The significance threshold was set at *P* < 0.05. Survival curves were presented as a percentage of live mice observed at 12-hour intervals over 7 days. The Mantel-Cox log-rank test (χ^2^) was used for statistical analysis of survival curves, with differences considered significant at *P* < 0.05.

### Study approval.

The experiments were previously approved by the Animal Ethics Committee of the UFMG (Protocols 137/2012 and 136/2014).

### Data availability.

Underlying data are available in the Supporting Data Values file.

## Author contributions

LCRR, MMT, and DGS created the study design. LCRR, RDNA, CBRM, MEFS, LML, JPPB, DB, BGR, LDT, and ACR performed data acquisition. LCRR, MMT, VP, CXL, and DGS analyzed and interpreted data and performed statistical analysis. CBRM, RDNA, FAA, MMT, CTF, CXL, and DGS prepared the manuscript.

## Funding support

- Instituto Nacional de Ciência e Tecnologia (INCT) em dengue e interação microrganismo hospedeiro (Grant 465425/2014-3).
- Coordenação de Aperfeiçoamento de Pessoal de Nível Superior (CAPES).
- Conselho Nacional de Desenvolvimento Científico e Tecnológico (CNPq).
- FAPEMIG.

## Supplementary Material

Supplemental data

Supporting data values

## Version 1 12/08/2025

Electronic publication

## References

[1] Singer M (2016). The Third International Consensus Definitions for Sepsis and Septic Shock (Sepsis-3). JAMA.

[2] Shen X (2021). Targeting neutrophils in sepsis: from mechanism to translation. Front Pharmacol.

[3] Wigerblad G, Kaplan MJ (2023). Neutrophil extracellular traps in systemic autoimmune and autoinflammatory diseases. Nat Rev Immunol.

[4] Liu N (2022). The immunological function of CXCR2 in the liver during sepsis. J Inflamm (Lond).

[5] Passos GF (2004). Kinin B1 receptor up-regulation after lipopolysaccharide administration: role of proinflammatory cytokines and neutrophil influx. J Immunol.

[6] Souza DG (2004). Role of bradykinin B2 and B1 receptors in the local, remote, and systemic inflammatory responses that follow intestinal ischemia and reperfusion injury. J Immunol.

[7] Souza DG (2003). Role of the bradykinin B2 receptor for the local and systemic inflammatory response that follows severe reperfusion injury. Br J Pharmacol.

[8] Rex DAB (2022). A modular map of Bradykinin-mediated inflammatory signaling network. J Cell Commun Signal.

[9] Shen JK, Zhang HT (2023). Function and structure of bradykinin receptor 2 for drug discovery. Acta Pharmacol Sin.

[10] Othman R (2021). Kinins and their receptors as potential therapeutic targets in retinal pathologies. Cells.

[11] van den Berg M (2022). Hospital-related costs of sepsis around the world: a systematic review exploring the economic burden of sepsis. J Crit Care.

[12] Stavrou EX (2018). Factor XII and uPAR upregulate neutrophil functions to influence wound healing. J Clin Invest.

[13] Metzemaekers M (2020). Neutrophil chemoattractant receptors in health and disease: double-edged swords. Cell Mol Immunol.

[14] Paegelow I (2002). Migratory responses of polymorphonuclear leukocytes to kinin peptides. Pharmacology.

[15] Ehrenfeld P (2006). Activation of kinin B1 receptors induces chemotaxis of human neutrophils. J Leukoc Biol.

[16] Figueroa CD (2015). Kinin B1 receptor regulates interactions between neutrophils and endothelial cells by modulating the levels of Mac-1, LFA-1 and intercellular adhesion molecule-1. Innate Immun.

[17] Bertram C (2007). Comparison of kinin B(1) and B(2) receptor expression in neutrophils of asthmatic and non-asthmatic subjects. Int Immunopharmacol.

[18] Ehrenfeld P (2009). Kinin B1 receptor activation turns on exocytosis of matrix metalloprotease-9 and myeloperoxidase in human neutrophils: involvement of mitogen-activated protein kinase family. J Leukoc Biol.

[19] Nasseri S (2015). Kinin B1 receptor antagonist BI113823 reduces acute lung injury. Crit Care Med.

[20] Martin EL (2010). Phosphoinositide-3 kinase gamma activity contributes to sepsis and organ damage by altering neutrophil recruitment. Am J Respir Crit Care Med.

[21] Kuhr F (2010). Differential regulation of inducible and endothelial nitric oxide synthase by kinin B1 and B2 receptors. Neuropeptides.

[22] Cunha TM (2007). TNF-alpha and IL-1beta mediate inflammatory hypernociception in mice triggered by B1 but not B2 kinin receptor. Eur J Pharmacol.

[23] Lizama AJ (2015). Expression and bioregulation of the kallikrein-related peptidases family in the human neutrophil. Innate Immun.

[24] Henderson LM (1994). Assembly of contact-phase factors on the surface of the human neutrophil membrane. Blood.

[25] Ehrenfeld P (2018). Functional interrelationships between the kallikrein-related peptidases family and the classical kinin system in the human neutrophil. Biol Chem.

[26] Stuardo M (2004). Stimulated human neutrophils form biologically active kinin peptides from high and low molecular weight kininogens. J Leukoc Biol.

[27] Hotchkiss RS (2016). Sepsis and septic shock. Nat Rev Dis Primers.

[28] Peters van Ton AM (2018). Precision immunotherapy for sepsis. Front Immunol.

[29] Pesquero JB (2000). Hypoalgesia and altered inflammatory responses in mice lacking kinin B1 receptors. Proc Natl Acad Sci U S A.

[30] Borkowski JA (1995). Targeted disruption of a B2 bradykinin receptor gene in mice eliminates bradykinin action in smooth muscle and neurons. J Biol Chem.

[31] Lima CX (2015). Therapeutic effects of treatment with anti-TLR2 and anti-TLR4 monoclonal antibodies in polymicrobial sepsis. PLoS One.

[32] Souza DG (2002). Increased mortality and inflammation in tumor necrosis factor-stimulated gene-14 transgenic mice after ischemia and reperfusion injury. Am J Pathol.

[33] Xu L (2013). Effects of adiponectin on acute lung injury in cecal ligation and puncture-induced sepsis rats. J Surg Res.

[34] Rios-Santos F (2007). Down-regulation of CXCR2 on neutrophils in severe sepsis is mediated by inducible nitric oxide synthase-derived nitric oxide. Am J Respir Crit Care Med.

[35] Schulze-Topphoff U (2009). Activation of kinin receptor B1 limits encephalitogenic T lymphocyte recruitment to the central nervous system. Nat Med.

[36] Alves-Filho JC (2006). Toll-like receptor 4 signaling leads to neutrophil migration impairment in polymicrobial sepsis. Crit Care Med.

