# Supplementary material for: BDKRB1 activation induces CXCR2 desensitization in neutrophils during severe sepsis and exacerbates disease severity
Source: JCI Insight. 2025 Dec 8;10(23):e185743. doi: 10.1172/jci.insight.185743 (PMC12890472; doi:10.1172/jci.insight.185743)
Supplement: Supplemental data [file jciinsight-10-185743-s200.pdf]

Table S1

Cytokine production in the lavage fluid and lung tissues of both WT and *Bdkrb1*$^{-/-}$ mice subjected to cecal ligation and puncture (CLP).

Cytokine (pg/mL)	Lavage				Lung			
	WT Sham	WT CLP	*B1r*$^{-/-}$ Sham	*B1r*$^{-/-}$ CLP	WT Sham	WT CLP	*B1r*$^{-/-}$ Sham	*B1r*$^{-/-}$ CLP
**CXCL2**	116 ± 21,29	11946 ± 2190*	94,48 ± 7,183	11170 ± 1579*	224,3 ± 88,65	5024 ± 210,8*	249,8 ± 62,33	2715 ± 245*#
**IL-1β**	0 ± 0	436,8 ± 92,67*	0 ± 0	424 ± 88,57*	796,6 ± 144	4413 ± 399,5*	697,7 ± 48,52	4382 ± 724,8*
**IL-6**	24,93 ± 2,901	3524 ± 701,4*	24,95 ± 5,423	1228 ± 290,8#	658,3 ± 109,1	2130 ± 402,5*	374,6 ± 42,23	828,6 ± 191,6#
**IL-10**	112 ± 18,76	3514 ± 786,5*	162,5 ± 36,29	3220 ± 456,6*	729,5 ± 40,8	2378 ± 188,2*	847,5 ± 89,8	2283 ± 477,2*

Values are expressed as mean ± SEM of at least five animals per group. *p < 0.05, when compared with sham group and #p < 0.05, when compared with wild-type animals submitted to CLP
